# The Diagnosis of Hepatitis and Hepatalgia

**Published:** 1845-06

**Authors:** 


*The Diagnosis of Hepatitis and Hepatalgia.*—The following summary, though it has nothing novel to recommend it, may be advantageously borne in mind:—

"Although the pains accompanying hepatalgia may be as intense as those of hepatitis, and in many instances, perhaps, more urgent, they are not constant, but are at the outset, and frequently, also, during the whole progress of the disorder, paroxysmal, affording in the interval a complete immunity from pain. The pathognomonic signs indicative of inflammatory action of the liver, are pyrexia, tumefaction, great tenderness in the hypochondrium, frequent and strong pulse, thirst, furred tongue, and vomiting, sometimes of a bilious, and at other times of a dark-coloured, secretion, as the substance of the liver more or less partakes of the invading disease. The bowels are irregular in their action, the evacuations presenting a great variety of appearances, according as the biliary secretion is more or less affected, and the urine is scanty and high-coloured. In hepatalgia, on the contrary, these signs are invariably wanting ; there may exist, indeed, constant pain and tenderness over the region of the liver, increased to a certain degree by pressure, but manifest exacerbations, even in the worst cases, occur, which are sufficiently indicative of its paroxysmal character. The functions of the organ may proceed uninterruptedly as in its healthy condition. The tongue may be quite clean, or sometimes, in the centre, there may be a gentle creamy fur, and the urine is generally increased in quantity, and is of a lighter colour than ordinary." *Treatment of Hepatalgia.*—"Gentle purgatives, combined with colchicum, ipecacuanha, and hyoscyamus, will seldom fail to work a speedy cure ; and if the constitution have suffered from protracted, unmitigated pain, alkaline vegetable tonics will effect that which we might in vain expect from the rough, insoluble, mineral preparations."—*Dr. Allnatt, Medical Gazette.*

